# What can we learn about stress and sleep from COVID-19 pandemic—perspective from the theory of preventive stress management

**DOI:** 10.3389/fpubh.2024.1383966

**Published:** 2024-04-04

**Authors:** Fang Liu, Weijie Liang, Hanqi Li, Yuyang Li, Yue Zhang, Lei Ding, Qianqian Zhang, Liang Chen

**Affiliations:** ^1^College of Teacher Education, Ningbo University, Ningbo, Zhejiang, China; ^2^Research Center for Psychological Development, University of Science and Technology Liaoning, Anshan, Liaoning, China

**Keywords:** COVID-19 pandemic, perceived social support, coping tendency, hope, stress, sleep quality

## Abstract

**Background:**

The COVID-19 pandemic has presented unique challenges to individuals worldwide, with a significant focus on the impact on sleep. However, the precise mechanisms through which emotional and cognitive variables mediate this relationship remain unclear. To expand our comprehensive understanding of variables, the present study utilizes the Preventive Stress Management theory, to test the relationship between perceived social support and sleep quality, as well as the effect of perceived COVID-19 stress, hope, negative emotions and coping styles.

**Methods:**

Data were collected in March 2022 from 1,034 college students in two universities located in Liaoning Province, China, using an online survey platform regarding perceived social support, perceived COVID-19 stress, sleep quality, hope, negative emotions and coping styles. The moderated mediation model were conducted using Process macro program (Model 6) and the syntax in SPSS.

**Results:**

The results revealed perceived COVID-19 stress and negative emotions sequentially mediated the negative relationship between perceived social support and sleep quality. Furthermore, hope and coping styles were found to moderate the sequential mediating effect.

**Conclusion:**

The present study sheds light on the pathways that affect sleep quality among college students during the COVID-19 pandemic. Findings highlight the protective roles played by positive social and personal resources, such as perceived social support, hope, and effective coping styles, against sleep problems. These insights have important implications for the development of targeted interventions to improve sleep outcomes during this challenging time.

## 1 Introduction

The COVID-19 pandemic has had profound health impacts globally, one of which is the significant rise in sleep issues. This concern has garnered considerable attention from researchers, with multiple studies highlighting the strong link between perceived stress from the pandemic and compromised sleep quality (1–3). Furthermore, certain investigators have delved into the potential mechanisms underlying this association (4, 5). However, a gap remains in the literature, as these studies often overlook the theoretical perspectives that could offer deeper insights into the intricate relationships among these variables.

The Theory of Preventive Stress Management (TPSM) (6–8) provides a comprehensive framework for understanding how perceived stress and its effects can be reduced during a pandemic outbreak. This theory outlines three distinct stages: stress generation, stress response, and consequences. By applying different preventive measures at each stage, we can effectively mitigate perceived stress, the stress response, and its consequences. These measures can be grouped into primary, secondary, and tertiary prevention (9, 10). Applying TPSM to the COVID-19 pandemic, primary prevention aims to intervene in COVID-19-related stressors, focusing mainly on the causes of stress; secondary prevention aims to intervene in stress responses, focusing mainly on individual actions and coping; and tertiary prevention aims to intervene in the final outcomes of stress, such as treating poor COVID-19 outcomes. In the present study, we focus on primary and secondary prevention, specifically examining how perceived social support, coping styles, and trait hope can counteract perceived COVID-19 stress.

According to TPSM, stressors are defined as physiological and psychological needs that triggers stress responses in individuals (6, 9, 11). This diverges from the conventional conception of stressors as merely stimulus events. Rather, it aligns with the initial assessment of stimulus events in cognitive appraisal theory (12). According to this theory, individuals determine whether a stimulus event affects them or not, and if it is perceived as harmful, it is considered a stressor. Hence, stressors are actually perceived stress. Perceived stress can be affected by two levels of factors; first, the frequency and intensity of the stimulus event, the more frequently the stimulus event occurs and the greater the intensity, the higher the level of stress perceived by the individual; and second, individual differences, evaluations of the stimulus event may vary from person to person, and for the same stimulus event, some people may perceive stress, while others may not, or there may be a level of difference.

During the COVID-19 pandemic, perceived COVID-19 stress among college students may stem from the following areas: academic (concerns about grades, rankings, and the future), health (concerns and fears about illness), interpersonal (difficulty achieving social closeness and may worry about friends or family), financial (debt and expenses), and family life (e.g., worrying about missing every phone call from parents) (13). Many of these aspects can be mitigated by social support. For example, family support will go a long way in directly alleviating financial stress, while help from classmates and friends may go a long way in alleviating difficulties in academic and interpersonal areas. In addition, support from others implies information about the good status of others, which alleviates individuals' worries about others. Therefore, we propose Hypothesis 1a: Perceived social support is negatively related to perceived COVID-19 stress.

Besides social support, personal traits, such as trait hope, can also influence how people perceive stress. According to Snyder's hope theory (14, 15), hope is a cognitive structure with two aspects: pathway thinking and agency thinking (15, 16). Pathway thinking is thinking about strategies to achieve a goal; for a highly hopeful person pursuing a specific goal, path thinking means identifying a feasible path and having confidence in that path. Agency thinking, on the other hand, is the motivational component of hope, which represents an individual's ability to use his or her own path to reach a desired goal (15). Stress arises when a particular situation threatens to reach a goal (17). In the case of the COVID-19 pandemic, pandemic may threaten college students' achievement of academic goals, interpersonal goals, thus causing stress. However, not all individuals will experience the same stress, hope theory suggests that individuals with high trait hope may be less likely to perceive these obstacles as stressful compared to individuals with low trait hope (15). In a COVID-19-related study, Gallagher et al. (18) also found that higher trait hope was associated with greater sense of wellbeing and perceived emotional control, as well as lower levels of anxiety and perceived COVID-19 stress, additionally, trait hope had an indirect effect on all outcomes through perceived emotional control. Therefore, we propose Hypothesis 1b: Trait hope is negatively associated with perceived COVID-19 stress.

In addition, trait hope may also amplify the role of social support. High hopefuls have a positive bias (15) and therefore may overestimate the effectiveness of treatment (19). As for the effect on perceived stress, overestimating the effectiveness of social support may contribute to reducing perceived COVID-19 stress. Therefore, we propose Hypothesis 1c: Trait hope will interact with social support to amplify the effect of social support on perceived stress.

TPSM posits that perceived stress leads to two types of responses (6). One is eustress, which is a positive, healthy response that leads to motivation and challenge (e.g., scientific pressure may stimulate a researcher's potential); the second is distress, which is a negative response that may result from a lack of stimulation (e.g., boredom), or it may result from a stress response that is too frequent, intense, or prolonged (e.g., anxiety). In current study, we focus on distress and use negative affect as measured by the Depression Anxiety Stress Scale (DASS-21). This scale is a self-reported questionnaire containing 21 questions designed to measure the extent of three negative affective states: depression, anxiety, and stress. Subscale of depression focuses on low mood, motivation, and self-esteem, and subscale of anxiety focuses on physiological activation, perceived panic, and fear, while subscale of stress focuses on tension and irritability (20). Thus, this scale provides a more comprehensive description of the symptoms of distress.

A large number of studies have now validated the positive correlation between perceived COVID-19 stress and negative emotions [e.g., (21–27)]. In addition, according to cognitive appraisal theory, assessing events as harmful leads to negative emotional responses (12). Therefore, we propose Hypothesis 2a: Perceived COVID-19 stress is positively correlated with negative emotions.

According to TPSM, the higher the perceived stress, the more likely an individual is to use passive coping rather than active coping (6, 28), and therefore, an individual's stress response can be moderated through secondary prevention. Secondary prevention focuses on several positive coping strategies, including emotion regulation and cognitive behavioral therapy (e.g., relaxation techniques, meditation techniques, hypnosis, and biofeedback training), faith and religion-based practices, emotional expression, exercise and wellness programs (6, 9, 14). Therefore, the role of coping styles may be consistent with secondary prevention.

At a more specific mechanistic level, the moderating effect of coping styles between perceived COVID-19 stress and negative emotions can be explained by cognitive appraisal theory. Cognitive appraisal theory suggests that individuals have three levels of appraisal of stimulus events. Among them, a primary appraisal is the individual's assessment of the relationship between the stimulus event and their interests, which is directly related to the perceived stress level. Secondary evaluation is the individual's assessment of the regulation and control of their response behavior, which is mainly related to whether people can control the stress events and the degree of control. When individuals assess their resources as insufficient or uncontrollable, individuals will experience negative emotions. Tertiary evaluation refers to the individual's assessment of the effectiveness and appropriateness of their emotional and behavioral responses. Negative emotions may arise when individuals perceive that their behavior is not effective enough (12). In this context, active and passive coping styles may have three opposite effects: first, active coping styles may increase an individual's resources, while passive coping styles do not; second, active coping styles may make individuals more inclined to assess stressors as controllable, while the opposite is true for passive coping styles; and third, active coping styles are more likely to be perceived as effective, whereas passive coping styles are not. These disparities underscore the critical role of coping styles in mediating the relationship between perceived stress and negative emotions. Therefore, we propose Hypothesis 2b: Coping styles can play a moderating role between perceived COVID-19 stress and negative emotions.

The TPSM suggests that when we experience distress, it can cause various behavioral, psychological, and medical problems (9). One of the psychological problems often associated with distress is sleep disturbance (6). There has been much literature exploring the relationship between negative emotions and sleep. For instance, Baglioni et al. (29) reviewed the connection between emotions and insomnia and identified different models of insomnia. In essence, when our thoughts are active, our emotions become heightened, and our body becomes activated, making it difficult to fall asleep (29). Empirical evidence suggests that this basic model is reliable [e.g., (30, 31)] and has been well-observed in the COVID-19 pandemic [e.g., (1, 27, 32–34)]. Therefore, we propose Hypothesis 3: Negative emotions are positively associated with poor sleep quality. However, the tertiary interventions mainly refer to therapeutic interventions for symptom consequences, which were beyond the scope of this study.

**Figure 1:**
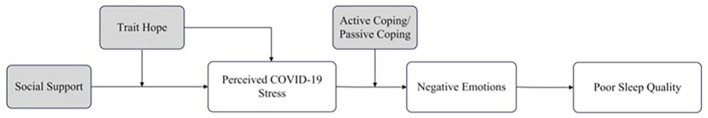
Conceptual framework of the relationship among perceived COVID-19 stress, negative emotions, poor sleep quality, perceived social support, trait hope, and coping styles.

Although numerous studies have examined the association between perceived stress and sleep quality during the COVID-19 pandemic, as well as the individual impacts of social support, personal traits, and coping styles, there remains a dearth of research that integrates these variables within a unified theoretical framework. Drawing upon the TPSM model, the present study provides a conceptual framework that explains the roles played by perceived social support, trait hope, and coping styles in the triadic relationship between perceived COVID-19 stress, emotions, and sleep quality (Figure 1 illustrates the conceptual framework). First, we posit that perceived social support and trait hope can directly reduce perceived COVID-19 stress, which in turn reduces negative emotions and sleep disturbances, which is ultimately expressed as a chain mediation. Second, we argue that hope amplifies the effects of social support on perceived COVID-19 stress. Finally, we argue that active coping styles reduce the effects of perceived stress on negative emotions, whereas passive coping styles amplify the effects of perceived stress on negative emotions.

## 2 Measures and methods

### 2.1 Participants

The participants were selected randomly from two universities located in Liaoning Province. A total of 1,034 university students participated in the study. All students were ranging from the first to third grades at the University of Science and Technology Liaoning. The data collection employed an online survey in March 2022. To ensure data uniqueness and validity, a unique electronic measurement network link was provided, limiting participants to submit only one survey per IP address. Following the exclusion of invalid questionnaires (including straight-lining and non-differentiation response patterns, as well as questionnaires with missing values), the final analysis included 980 participants (630 men and 350 women) aged between 17 and 29 years (M = 19.43, SD = 1.14). Written informed consent was obtained from all students and their affiliated universities. The study design was approved by the Human Research Ethics Committee of local university of the corresponding author. Table 1 provided the demographic profiles of the participants.

**Table 1:** Descriptive statistics and correlation coefficient matrix (*N* = 980).

	**1**	**2**	**3**	**4**	**5**	**6**	**7**	**8**	**9**
1. Gender	–								
2. Age	−0.181^***^	–							
3. PCOS	0.010	−0.017	–						
4. NE	−0.066^*^	0.033	0.503^***^	–					
5. PSQI	0.022	0.082^*^	0.305^***^	0.531^***^	–				
6. PSS	0.099^**^	−0.096^**^	−0.132^***^	−0.254^***^	−0.201^***^	–			
7. TH	0.062	−0.045	−0.076^*^	−0.182^***^	−0.195^***^	0.466^***^			
8. ACS	0.081^*^	−0.059	0.051	−0.157^***^	0.123^***^	0.486^***^	0.647^***^	-	
9. PCS	−0.052	−0.017	0.133^***^	0.234^***^	0.125^***^	0.033	0.228^***^	0.340^***^	
*M*	–	19.430	2.310	11.670	3.299	59.760	22.201	1.847	1.395
*SD*	–	1.139	0.576	12.604	2.638	14.887	4.095	0.570	0.605

^*^p < 0.05, ^**^p < 0.01, ^***^p < 0.001. Gender is coded as a categorical variable (male = 0, female = 1).

PCOS, perceived COVID−19 stress; NE, negative emotions; PSQI, Pittsburgh Sleep Quality Index; PSS, perceived social support; TH, trait hope; ACS, active coping style; PCS, passive coping style; M, Means; SD, Standard deviation.

Based on our planned statistical analyses, we calculated the required sample size. In view of the fact that there seemed not a completely suitable estimation method in a two-stage moderated mediation model (35), we refer to previous studies and utilized the Monte Carlo Power Analysis for Indirect Effects technique developed by Schoemann et al. (36) to determine the minimum sample size needed for this study. With guidance from correlations and standard deviations from previous studies (5, 37, 38), and assuming 80% power in a two-stage mediation model, we calculated a minimum requirement of 650 participants. Given that our sample size far exceeds 650, we believe that the sample size should be sufficient.

### 2.2 Measures

#### 2.2.1 Perceived COVID-19 stress

Perceived COVID-19 stress was evaluated by the COVID-19 Stress Questions (21). This questionnaire comprises eight items, such as “How likely is it that you could become infected with the COVID-19 virus?”. The items range from 1 (not at all) to 4 (very much). Higher scores indicate higher levels of perceived COVID-19 stress. (α = 0.81).

#### 2.2.2 Negative emotions

Negative emotions were evaluated by the short form of Depression, Anxiety and Stress Scale (DASS-21) (39). The scale comprises three subscales: depression (seven items, e.g., “I felt that life was meaningless”), anxiety (seven items, e.g., “I was worried about situations in which I might panic and make a fool of myself”), and stress (seven items, e.g., “I was intolerant of anything that kept me from getting on with what I was doing”). The items range from 0 (never) to 3 (always). Higher scores indicate higher levels of negative emotions experienced by the participants. (α = 0.97).

#### 2.2.3 Poor sleep quality

Poor sleep quality was evaluated by the Chinese version of the Pittsburgh Sleep Quality Index (PSQI) (40). The scale consists of 19 items that encompass seven factors: subjective sleep quality, sleep latency, sleep duration, habitual sleep efficiency, sleep disturbances, use of sleep medication, and daytime dysfunction. PSQI scores range from 0 to 21, with higher scores indicating poorer sleep quality (α = 0.88).

#### 2.2.4 Perceived social support

Perceived social support was evaluated by the Multidimensional Perceived Social Support Scale (MSPSS) (41). This scale comprises three subscales: family (four items, e.g., “My family really try to help me”), friends (four items, e.g., “My friends really try to help me”), and significant others (four items, e.g., “There is a special person who is around when I am in need”). The items range from 1 (definitely disagree) to 7 (definitely agree). Higher scores indicate higher levels of perceived social support (α = 0.97).

#### 2.2.5 Trait hope

Trait hope was evaluated by the Trait Hope Scale (14). This scale comprises two subscales: pathways thinking (four items, e.g., “I can think of many ways to get out of jams”), and agency thinking (four items, e.g., “I energetically pursue my goals”). The items range from 1 (definitely false) to 4 (definitely true). Higher scores indicate higher levels of trait hope (α = 0.88).

#### 2.2.6 Coping styles

Coping styles were evaluated by the Simplified Coping Style Questionnaire (SCSQ) (42). The SCSQ comprises 20 items separated into passive (eight items, e.g., “I try to forget the whole thing”) and active coping styles (12 items, e.g., “I could try to look on the bright side of things”) (42). The items range from 0 (never) to 3 (always), with higher scores indicating greater active/passive coping. (α = 0.91, 0.93, 0.85 for total score, active coping, passive coping, respectively).

#### 2.2.7 Statistical analysis

We employed ordinary least squares (OLS) regression models to test the effects of interested variables on perceived COVID-19 stress, negative emotions and poor sleep quality. Then we ran a serial mediation model (using model 6 in SPSS PROCESS macro v 4.2) (43) to explore the proposed mediation relationship. Finally, we employed a conditional moderated mediation analysis to examine the moderating role of trait hope and coping styles in the mediation process (using syntax in SPSS), the syntax was “process y=ZPSQI/m=ZCS ZDASS/x=ZPSSS/w=Zhope/Z=CT/cov= age gender/conf=95.0/boot=5000/plot=1/moments=1/total=1/bmatrix=1,1,1,1,1,1/wmatrix=1,1,1,1,1,1/zmatrix=1,1,1,1,1,1.”. The standardization was applied to all variables of interest in the analysis due to the presence of interaction terms. We controlled for age and gender in the subsequent analysis. The figure s in the text were produced using sangerbox.com, Excel.

## 3 Results

**Figure 2:**
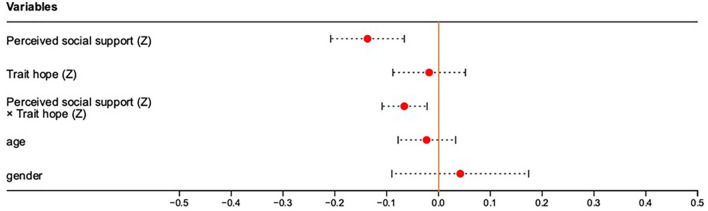
Regression plot of the Perceived social support (Z), Trait hope (Z), Perceived social support (Z) × Trait hope (Z), age and gender predicting Perceived COVID-19 stress (Z). (Z) is the result after standardization.

**Figure 3:**
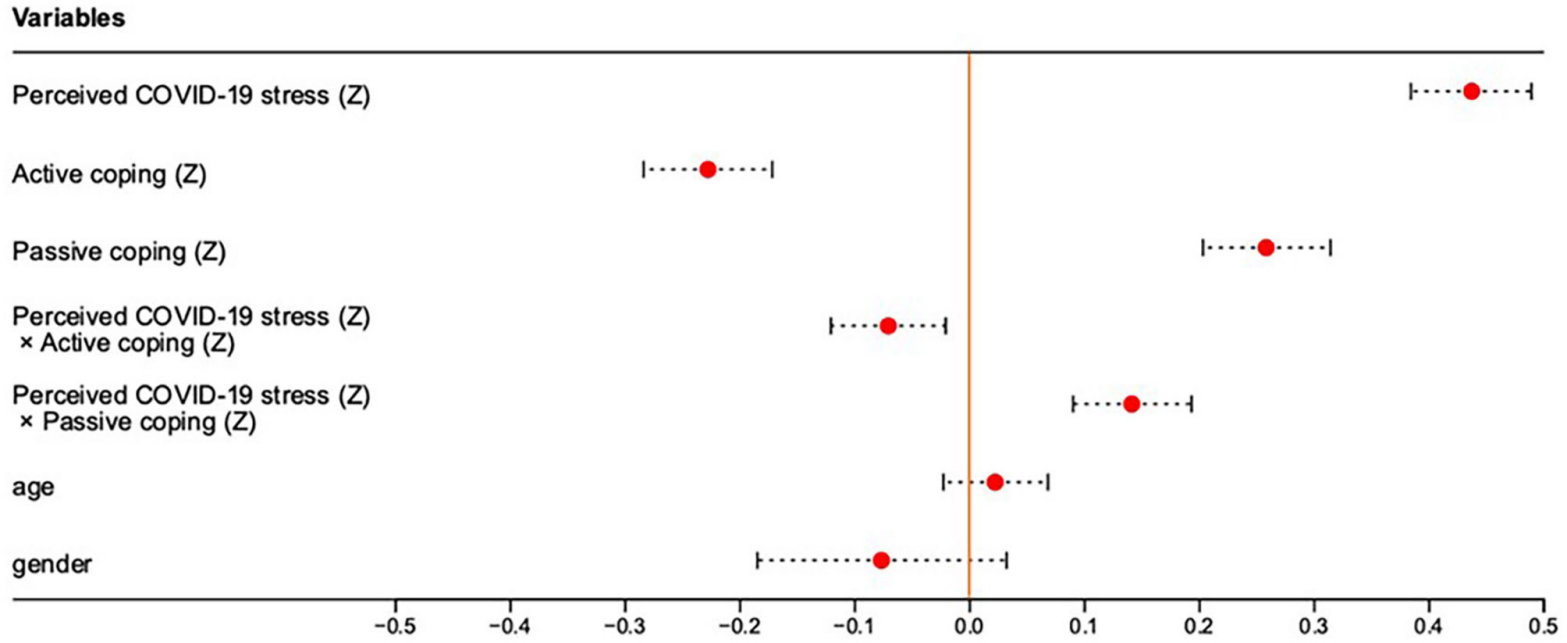
Regression plot of the Perceived COVID-19 stress (Z), Active coping (Z), Passive coping (Z), Perceived COVID-19 stress (Z) × Active coping (Z), Perceived COVID-19 stress (Z) × Passive coping (Z), age and gender predicting Negative emotions (Z). (Z) is the result after standardization.

**Figure 4:**
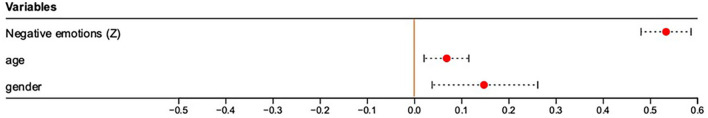
Regression plot of the Negative emotions (Z), age and gender predicting Poor sleep quality (Z). (Z) is the result after standardization.

**Figure 5:**
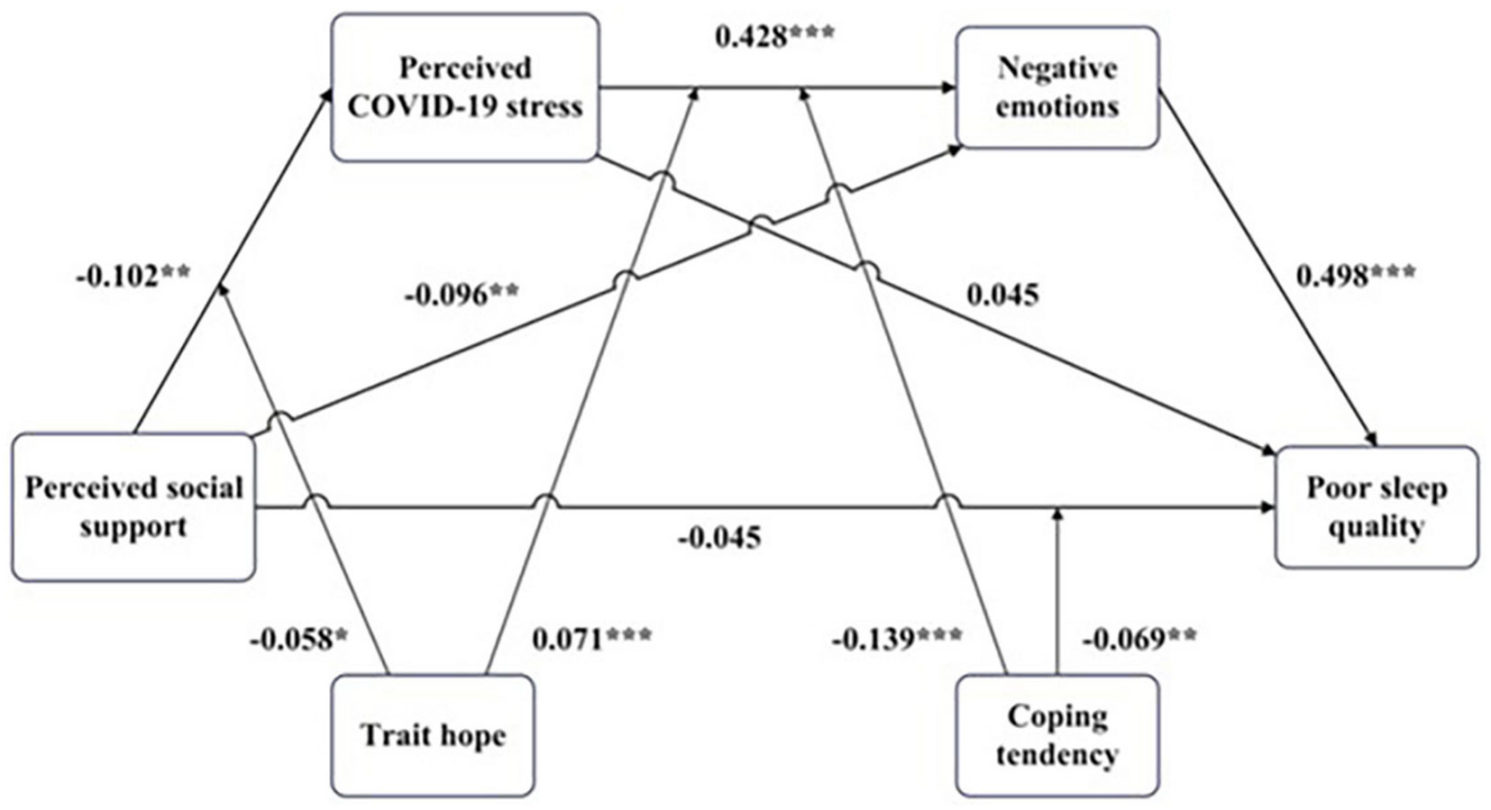
Illustrated moderated mediation of perceived social support, perceived COVID-19 stress, negative emotions, poor sleep quality, trait hope, and coping tendency. Bootstrap resample = 5,000. Statistical controls include age, gender. ^*^p < 0.05, ^**^p < 0.01, ^***^p < 0.001.

Table 1 presents descriptive statistics and correlations for each variable. In the first step, we ran regression analyses to test hypotheses initially. The results of the OLS regression are presented in Supplementary Table S1–S3 (see Figures 2–5 for more details). The results suggested that: First, those who perceive more social support were likely to perceive less COVID-19 stress (B = −0.137, SE = 0.036, *p* < 0.001); and people with high trait hope were likely to gain more benefits from perceived social support (B = −0.066, SE = 0.022, *p* = 0.003); Second, those who perceived more COVID-19 stress were likely to experience higher negative emotions (B = 0.437, SE = 0.027, *p* < 0.001); Those who employed more active coping styles were likely to experience lower negative emotions (B = −0.228, SE = 0.028, *p* < 0.001) and gain negative emotions from perceived COVID-19 stress (B = −0.071, SE = 0.025, *p* = 0.005); Those who employed more passive coping styles were likely to experience higher negative emotions (B = 0.258, SE = 0.028, *p* < 0.001) and gain higher negative emotions from perceived COVID-19 stress (B = 0.141, SE = 0.026, *p* < 0.001); Then those who experienced higher negative emotions were likely to experience poorer sleep quality (B = 0.533, SE = 0.027, *p* < 0.001); Those who were older were likely to experience poorer sleep quality (B = 0.068, SE = 0.024, *p* = 0.005); Girls were likely to experience poorer sleep quality compared to boys (B = 0.149, SE = 0.057, *p* = 0.009). Next, to explore whether here is a serial mediation (perceived social support → perceived COVID-19 stress → negative emotions → poor sleep quality), we ran mediation analyses using the model 6 in SPSS PROCESS macro v 4.2 (43). The bootstrapping method was used to estimate the indirect effects (*N* = 5,000).

The results are illustrated in Figure 5. As observed, we found that perceived social support could affect poor sleep quality in three ways: Ind3: more perceived social support → less perceived COVID-19 stress → lower negative emotions → poorer sleep quality [B = −0.022, SE = 0.010, 95% CI: (−0.042, −0.003)]; Ind2: more perceived social support → lower negative emotions → poorer sleep quality [B = −0.048, SE = 0.018, 95% CI: (−0.084, −0.015)]; Ind1: higher perceived social support → poorer sleep quality [B = −0.045, SE = 0.032, 95% CI: (−0.108, 0.018)]. These results indicated that perceived social support affect poor sleep quality by many ways, and the strongest way may be that perceived social support makes people experience negative emotions less. And the effect of perceived COVID-19 stress on poor sleep quality was total mediated by negative emotions.

Finally, we explored the moderating role of trait hope and coping styles on each path of the mediation process. Though, from the regression analyses, it is already known that trait hope could moderate the effect of perceived social support on perceived COVID-19 stress, and the coping styles could moderate the effect of perceived COVID-19 stress on negative emotions, we did not know whether they have a moderating effect on other paths, and how these moderating effects influence the final outcome. To analysis the moderating effect on each path, we employed conditional process analyses using the SPSS syntax with PROCESS macro (43) for 2 conditional moderators. As process only allow two moderators, we transformed active coping style and passive coping style into coping tendency, which is determined by “Z _activecoping_ – Z _passivecoping_” (44). The higher the value of this indicator, the more inclined the individual is to use active coping styles. Although this may result in the loss of some information characteristics, we could get a rough result, and some articles had employed this indicator (44).

The general results are illustrated in Figure 5, only the significant moderating effects were plotted. As observed, except for the two moderating effects that we originally assumed, we found that people with higher trait hope could gain more negative emotions from perceived COVID-19 stress. We also found that coping tendency could aggregate the direct effect of perceived social support on poor sleep quality, those who prefer to use active coping styles were likely to gain better sleep quality from perceived social support. The bootstrap results for the moderated mediation effects were shown in Table 2 (direct effect) and Supplementary Table S4, S5 (Table 2 describes the overall effect of trait hope and coping style on the process from perceived social support to sleep quality, while Supplementary Table S4, S5 describe the effect of trait hope and coping style on the stage process from perceived social support to perceived stress, and perceived stress to negative emotion.). The plots of interaction effects were shown in Supplementary Figures S1–S3.

**Table 2:** Conditional effects of perceived social support on poor sleep quality through perceived COVID-19 stress and negative emotions at different levels of trait hope and coping tendency.

**Trait hope level and coping tendency level**	**Effect**	**Boot SE**	**LLCI**	**ULCI**
**Direct effect**				
Low (−1 SD) trait hope and low (−1 SD) coping tendency	0.055	0.040	−0.023	0.133
Low (−1 SD) trait hope and mean coping tendency	−0.024	0.038	−0.098	0.050
Low (−1 SD) trait hope and high (+1 SD) coping tendency	−0.103	0.056	−0.212	0.006
Mean trait hope and low (−1 SD) coping tendency	0.034	0.041	−0.046	0.115
Mean trait hope and mean coping tendency	−0.045	0.032	−0.108	0.018
Mean trait hope and high (+1 SD) coping tendency	−0.124	0.047	−0.216	−0.032
High (+1 SD) trait hope and low (−1 SD) coping tendency	0.014	0.052	−0.088	0.116
High (+1 SD) trait hope and mean coping tendency	−0.066	0.039	−0.143	0.012
High (+1 SD) trait hope and high (+1 SD) coping tendency	−0.135	0.047	−0.237	−0.052
**Indirect effect 1**				
Low (−1 SD) trait hope and low (−1 SD) coping tendency	−0.002	0.005	−0.014	0.007
Low (−1 SD) trait hope and mean coping tendency	−0.003	0.006	−0.018	0.006
Low (−1 SD) trait hope and high (+1 SD) coping tendency	−0.005	0.010	−0.032	0.012
Mean trait hope and low (−1 SD) coping tendency	−0.003	0.006	−0.017	0.008
Mean trait hope and mean coping tendency	−0.005	0.004	−0.014	0.002
Mean trait hope and high (+1 SD) coping tendency	−0.007	0.007	−0.024	0.004
High (+1 SD) trait hope and low (−1 SD) coping tendency	0.001	0.012	−0.027	0.023
High (+1 SD) trait hope and mean coping tendency	−0.003	0.008	−0.020	0.012
High (+1 SD) trait hope and high (+1 SD) coping tendency	−0.006	0.009	−0.026	0.010
**Indirect effect 2**				
Low (−1 SD) trait hope and low (−1 SD) coping tendency	−0.047	0.025	−0.102	−0.003
Low (−1 SD) trait hope and mean coping tendency	−0.040	0.023	−0.088	0.001
Low (−1 SD) trait hope and high (+1 SD) coping tendency	−0.032	0.031	−0.101	0.023
Mean trait hope and low (−1 SD) coping tendency	−0.055	0.025	−0.106	−0.007
Mean trait hope and mean coping tendency	−0.048	0.018	−0.084	−0.015
Mean trait hope and high (+1 SD) coping tendency	−0.041	0.022	−0.088	−0.002
High (+1 SD) trait hope and low (−1 SD) coping tendency	−0.061	0.031	−0.122	−0.001
High (+1 SD) trait hope and mean coping tendency	−0.055	0.022	−0.099	−0.012
High (+1 SD) trait hope and high (+1 SD) coping tendency	−0.048	0.021	−0.094	−0.009
**Indirect effect 3**				
Low (−1 SD) trait hope and low (−1 SD) coping tendency	−0.008	0.017	−0.040	0.027
Low (−1 SD) trait hope and mean coping tendency	−0.008	0.012	−0.033	0.015
Low (−1 SD) trait hope and high (+1 SD) coping tendency	−0.006	0.010	−0.029	0.014
Mean trait hope and low (−1 SD) coping tendency	−0.026	0.018	−0.061	0.012
Mean trait hope and mean coping tendency	−0.022	0.010	−0.042	−0.003
Mean trait hope and high (+1 SD) coping tendency	−0.016	−0.010	−0.037	0.001
High (+1 SD) trait hope and low (−1 SD) coping tendency	−0.044	0.026	−0.095	0.009
High (+1 SD) trait hope and mean coping tendency	−0.037	0.014	−0.066	−0.009
High (+1 SD) trait hope and high (+1 SD) coping tendency	−0.028	0.011	−0.052	−0.009

Direct effect, perceived social support → poor sleep quality; Indirect effect 1: perceived social support → perceived COVID-19 stress → poor sleep quality; Indirect effect 2: perceived social support → negative emotions → poor sleep quality; Indirect effect 3: perceived social support → perceived COVID-19 stress → negative emotions → poor sleep quality.

The results of the conditional effects of perceived social support on poor sleep quality via four pathways at different levels (−1 SD, mean, and +1 SD) of trait hope and coping tendency are included in Table 2. We found complex results. In the direct effects of social support on poor sleep quality, the effect was significant only when the coping tendency was at a high level and the trait hope was at mean or high levels. In the direct effect 2 (higher perceived social support → lower negative emotions → poorer sleep quality), we found that the effect could be significant when both trait hope and coping tendency were at a low level, and the effects was always significant when trait hope was at a high level. In the indirect effect 3 (higher perceived social support → less perceived COVID-19 stress → lower negative emotions → poorer sleep quality), we found that only when both trait hope and coping tendency were at mean or high levels, the effects could be significant.

## 4 Discussion

### 4.1 Main findings

Previous studies have explored the possible mechanism in the relationship between perceived stress and sleep in the COVID-19 pandemic, however few studies have integrated the above variables. Based on the TPSM framework, current study explored the possible protective factors in relationship between perceived stress and sleep in the COVID-19 pandemic. Results indicated that high levels of perceived social support, trait hope, and coping style would decrease the negative effect of perceived COVID-19 stress on sleep during the pandemic.

Results showed that high levels of perceived social support can effectively reduce individual's perceived COVID-19 stress, and then reduce negative emotions and poor sleep quality, which is consistent with H1 a, H1 c, H2 a, and H3. In addition, we found that trait hope moderated the path from perceived social support to perceived COVID-19 stress, and coping styles moderated the path from perceived COVID-19 stress to negative emotions, which was consistent with H1b and H2b. However, our results did not support H1b, that is, we found that trait hope does not directly reduce perceived stress. Finally, when the focus shifted to the final impact of these moderating effects on poor sleep quality, the results showed that perceived social support could significantly reduce poor sleep quality only when trait hope was at a moderate or higher level and individuals were not inclined to use passive coping styles; the two exceptions are that when individuals with low trait hope and high trait hope tend to use negative strategies, perceived social support can also reduce poor sleep quality by directly reducing negative emotions.

From the perspective of TPSM, perceived social support can reduce perceived COVID-19 stress due to others can directly help deal with stressors. For example, economic support from families can directly reduce economic stress, and help from classmates and teachers can directly reduce academic stress (6, 9, 11). Our results also showed a certain boundary condition, that is, the influence of trait hope and coping style: people with high trait hope may amplify the perceived effectiveness of others' help. On the contrary, people with low trait hope may feel that the help from others is inefficient or ineffective. The individual's coping style is mainly used to reduce the negative stress response. Active coping styles can reduce the response of perceived stress to negative emotions, while passive coping styles are the opposite, which is consistent with the previous results (44).

However, we also found that high levels of perceived social support can directly reduce negative emotions and poor sleep quality, which may suggest that there are other mediating factors. One possibility is that perceived social support not only offsets perceived COVID-19 stress but also facilitates effective coping, which in turn motivate positive emotions. This can also explain why coping styles can moderate the direct path because active coping can promote and amplify responses to positive factors (45). Positive emotions not only offset negative emotions but also have their unique roles, such as increasing psychological resources (34) and promoting tolerance and patience (46).

We also found that in the path from perceived COVID-19 stress to negative emotions, high levels of trait hope amplify the effect of perceived COVID-19 stress on negative emotions, regardless of coping style. This showed that when an individual with high trait hope has perceived COVID-19 stress, will produce more negative emotions. This can be explained by the hope theory. If an individual perceives stress, it means that the current situation is beyond control to a certain extent, and people with high trait hope may have higher expectations of the situation. When expectations are broken, the individual's psychological state will weaken (47). However, the hope theory also holds that even if hopeful people find their hopes dashed, they will not be defeated, but try another effective strategy to pursue their goals. As a result, hopeful people re-actively think when faced with obstacles (15), while low hopeful people tend to be frustrated and lethargic when faced with obstacles, especially in terms of behavior. As reflected in this article, the hopefuls are more likely to adopt active coping styles than their counterpart (see Table 1). Active coping styles will reduce the impact of perceived stress on negative emotions. Therefore, present results may reflect the dynamic pattern of high trait hope in coping with stress.

Finally, our results support the framework provided by TPSM, and we verify the role of perceived social support as primary prevention and the role of coping style as secondary prevention. This largely illustrates the importance of classical theory in clarifying the relationship between variables. The framework provides beneficial insights, which give a comprehensive relationship between variables. Even if the current study is only a cross-sectional study, it can provide a lot of valuable information.

### 4.2 Practical implications

Current findings are particularly important in the context of public health, showing the different roles of external support and internal response in responding to public health crises, and revealing the unique impact of individual differences in these processes. First, in the discussion of the COVID-19 pandemic, some articles have argued that the COVID-19 pandemic is uncontrollable for the general public (48), and subsequently may argue that perceived stressors are also unpredictable; however, in reality, only a portion of people's perceived stress stems from the fear of getting sick, and much more is the fear of the social, economic, and economic consequences derived from the COVID-19 pandemic. much more from concerns about social, economic, and academic aspects derived from the COVID-19 pandemic (13), the latter of which can offset by social support or otherwise. Second, hope is an important factor influencing the role of perceived social support on perceived stress. Although trait hope is measured in this paper, hope can also be state-based, and triggering state hope may elicit similar effects as trait hope. Since hope is goal-oriented, figuring out ways to evoke goals in life may help combat stress (49, 50), so individuals can de-stress by being committed to work-study (51), and the government can reduce feelings of hopelessness by providing opportunities in future. Finally, coping styles are also an important part of mitigating the consequences of stress, with positive coping styles helping to reduce an individual's stress response, while negative coping styles do the opposite. For example, when faced with a disaster, the public media could promote positive coping styles to help people deal with the current difficulties.

### 4.3 Limitations and future directions

This study is not without limitations. First, the reliance on cross-sectional data limits our understanding of the causal relationship between variables. For example, once stress is recognized and starts affecting emotions and sleep, even if the original stressors is removed, the sleep issues that have already emerged might result in continued stress, negative emotions, ineffective coping styles, and future sleep disturbances (52, 53). Failing to focus on any of these factors can prevent problems from being fully resolved. This is where the importance of TPSM lies. Unfortunately, current study involves neither tertiary interventions nor longitudinal research. Future studies can consider exploring these directions. Additionally, fluctuating variables over time could be captured more effectively using diary methods or experience sampling. Second, while this paper focuses on applying and expanding TPSM during the COVID-19 pandemic, there are other theories worth exploring for insights as outlined in Bhattacharjee et al.'s review (54). Future research can integrate theories to discuss the relationship between variables in depth. Third, the absence of positive emotions in both measurements and theoretical frameworks may affect the interpretation of the results. Positive emotions have unique roles beyond just offsetting negative emotions, as they can enhance resilience and transform negatives into positives (55). Future research could integrate positive emotions into frameworks for a comprehensive understanding. Fourth, factors such as stamina and fatigue may play a significant role in the relationship between sleep quality and perceived stress (56). Future research can consider including these variables. Finally, as college students served as the study's primary demographic, it is important to determine whether the results could be applied to people of different ages. Future research could validate the results by including participants from a broader range of ages. Moreover, as all information in this study was sourced from participants' subjective reports, there is a risk of reporting bias. Future research may take this into account by gathering information from various sources to increase the objectivity of the measurements.

## 5 Conclusion

This study employed the TPSM to examine the integration of perceived COVID-19 stress, negative emotions, poor sleep quality, and related resilience variables during the COVID-19 pandemic. Results suggested that perceived social support effectively alleviated perceived COVID-19 stress, negative emotions, and poor sleep quality. Furthermore, trait hope not only enhanced the positive effects of perceived social support, but enhanced the negative effects of perceived COVID-19 stress in situations where individuals already perceived stress. Additionally, active coping styles attenuated the transition from perceived COVID-19 stress to negative emotions, whereas passive coping styles had the opposite effect.

## Data availability statement

The raw data supporting the conclusions of this article will be made available by the corresponding authors.

## Ethics statement

The studies involving humans were approved by College of Teacher Education, Ningbo University. The studies were conducted in accordance with the local legislation and institutional requirements. Written informed consent for participation in this study was provided by the participants' legal guardians/next of kin.

## Author contributions

FL: Writing – original draft, Writing – review & editing. WL: Writing – original draft. HL: Writing – original draft. YL: Writing – original draft. YZ: Writing – review & editing. LD: Writing – review & editing. QZ: Writing – review & editing. LC: Writing – review & editing.
