# Supplementary material for: What can we learn about stress and sleep from COVID-19 pandemic—perspective from the theory of preventive stress management
Source: Front Public Health. 2024 Apr 4;12:1383966. doi: 10.3389/fpubh.2024.1383966 (PMC11024324; doi:10.3389/fpubh.2024.1383966)
Supplement: Supplementary file 1 [file Data_Sheet_1.docx]

Supplementary Material

# Supplementary Figures and Tables

## Supplementary Figures

**Supplementary Figure 1.** Conditional effects of perceived social support on perceived COVID-19 stress at different levels of trait hope and coping tendency.

**Supplementary Figure 2.** Conditional effects of perceived COVID-19 stress on negative emotions at different levels of trait hope and coping tendency.

**Supplementary Figure 3.** Conditional direct effects of perceived social support on poor sleep quality at different levels of trait hope and coping tendency.

## Supplementary Tables

Supplementary Table 1. Regression result of intercept, Perceived social support (Z), Trait hope (Z), Perceived social support (Z) × Trait hope (Z), age and gender predicting Perceived COVID-19 stress (Z).

| Variables | *B* | *t* | *95%CI* |
| --- | --- | --- | --- |
| intercept | 0.411 | 0.717 | -0.714 ~ 1.537 |
| Perceived social support (Z) | -0.137 | -3.801^***^ | -0.208 ~ -0.066 |
| Trait hope (Z) | -0.018 | -0.507 | -0.088 ~ 0.052 |
| Perceived social support (Z) × Trait hope (Z) | -0.066 | -2.963^**^ | -0.109 ~ -0.022 |
| age | -0.023 | -0.797 | -0.078 ~ 0.033 |
| gender | 0.042 | 0.628 | -0.09 ~ 0.174 |

Notes: ^*^*p* < 0.05, ^**^*p* < 0.01, ^***^*p* < 0.001. Gender is coded as a categorical variable (male = 0, female = 1). (Z) is the result after standardization.

Supplementary Table 2. Regression result of intercept, Perceived social support (Z), Active coping (Z), Passive coping (Z), Perceived social support (Z) × Active coping (Z), Perceived social support (Z) × Passive coping (Z), age and gender predicting Negative emotions (Z).

| Variables | *B* | *t* | *95%CI* |
| --- | --- | --- | --- |
| intercept | -0.355 | -0.753 | -1.280 ~ 0.570 |
| Perceived social support (Z) | 0.437 | 16.323^***^ | 0.384 ~ 0.489 |
| Active coping (Z) | -0.228 | -8.039^***^ | -0.284 ~ -0.172 |
| Passive coping (Z) | 0.258 | 9.141^***^ | 0.203 ~ 0.314 |
| Perceived social support (Z) ×  Active coping (Z) | -0.071 | -2.788^**^ | -0.121 ~ -0.021 |
| Perceived social support (Z) ×  Passive coping (Z) | 0.141 | 5.370^***^ | 0.09 ~ 0.193 |
| age | 0.022 | 0.966 | -0.023 ~ 0.068 |
| gender | -0.077 | -1.380 | -0.185 ~ 0.032 |

Notes: ^*^*p* < 0.05, ^**^*p* < 0.01, ^***^*p* < 0.001. Gender is coded as a categorical variable (male = 0, female = 1). (Z) is the result after standardization.

Supplementary Table 3. Regression result of intercept, Negative emotions (Z), age and gender predicting Perceived COVID-19 stress (Z).

| Variables | *B* | *t* | *95%CI* |
| --- | --- | --- | --- |
| intercept | -1.517 | -3.106^***^ | -2.476 ~ -0.559 |
| Negative emotions (Z) | 0.533 | 19.709^***^ | 0.384 ~ 0.489 |
| age | 0.068 | 2.811^**^ | 0.020 ~ 0.115 |
| gender | 0.149 | 2.600^**^ | 0.037 ~ 0.261 |

Notes: ^*^*p* < 0.05, ^**^*p* < 0.01, ^***^*p* < 0.001. Gender is coded as a categorical variable (male = 0, female = 1). (Z) is the result after standardization.

Supplementary Table 4. Conditional effects of perceived social support on perceived COVID-19 stress at different levels of trait hope and coping tendency.

| Trait hope level and coping tendency level | Effect | Boot SE | LLCI | ULCI |
| --- | --- | --- | --- | --- |
| Low (−1 SD) trait hope and low (−1 SD) coping tendency | -0.031 | 0.044 | -0.118 | 0.056 |
| Low (−1 SD) trait hope and mean coping tendency | -0.044 | 0.044 | -0.130 | 0.043 |
| Low (−1 SD) trait hope and high (+1 SD) coping tendency | -0.056 | 0.063 | -0.180 | 0.067 |
| Mean trait hope and low (−1 SD) coping tendency | -0.089 | 0.046 | -0.180 | 0.002 |
| Mean trait hope and mean coping tendency | -0.102 | 0.038 | -0.175 | -0.028 |
| Mean trait hope and high (+1 SD) coping tendency | -0.115 | 0.052 | -0.216 | -0.013 |
| High (+1 SD) trait hope and low (−1 SD) coping tendency | -0.147 | 0.060 | -0.264 | -0.030 |
| High (+1 SD) trait hope and mean coping tendency | -0.160 | 0.046 | -0.250 | -0.070 |
| High (+1 SD) trait hope and high (+1 SD) coping tendency | -0.173 | 0.051 | -0.274 | -0.072 |

Supplementary Table 5. Conditional effects of perceived COVID-19 stress on negative emotions at different levels of trait hope and coping tendency.

| Trait hope level and coping tendency level | Effect | Boot SE | LLCI | ULCI |
| --- | --- | --- | --- | --- |
| Low (−1 SD) trait hope and low (−1 SD) coping tendency | 0.517 | 0.037 | 0.443 | 0.590 |
| Low (−1 SD) trait hope and mean coping tendency | 0.357 | 0.035 | 0.289 | 0.425 |
| Low (−1 SD) trait hope and high (+1 SD) coping tendency | 0.197 | 0.053 | 0.094 | 0.300 |
| Mean trait hope and low (−1 SD) coping tendency | 0.588 | 0.036 | 0.518 | 0.658 |
| Mean trait hope and mean coping tendency | 0.428 | 0.027 | 0.376 | 0.481 |
| Mean trait hope and high (+1 SD) coping tendency | 0.268 | 0.044 | 0.183 | 0.354 |
| High (+1 SD) trait hope and low (−1 SD) coping tendency | 0.659 | 0.045 | 0.570 | 0.748 |
| High (+1 SD) trait hope and mean coping tendency | 0.499 | 0.033 | 0.434 | 0.564 |
| High (+1 SD) trait hope and high (+1 SD) coping tendency | 0.340 | 0.043 | 0.254 | 0.425 |
